# Osteoporotic Thoracolumbar Vertebral Fractures With Neurological Deficit Treated by Balloon Kyphoplasty Augmented with Newly Developed Minimally Invasive Posterior Hook Stabilization

**DOI:** 10.7759/cureus.20505

**Published:** 2021-12-18

**Authors:** Toshio Doi, Ryutaro Kozuma, Junichi Arima

**Affiliations:** 1 Orthopaedic Surgery, Hiroshima Red Cross Hospital & Atomic-bomb Survivors Hospital, Hiroshima, JPN

**Keywords:** neurological deficit, kyphosis, spinal instrumentation, vertebroplasty, balloon kyphoplasty, hook, minimal invasive surgery, osteoporotic vertebral fracture

## Abstract

Most osteoporotic vertebral fractures (OVFs) are treated conservatively, but surgery is often indicated for residual pain, neuropathy, or severe deformity. OVFs tend to develop in elderly patients, so less invasive surgery is desirable. Surgery is mainly performed to stabilize the fractured vertebral body. Percutaneous cement augmentation, such as via balloon kyphoplasty (BKP), has produced satisfactory results as a surgical method for managing OVFs. Posterior fixation with implants is often performed with or without cement augmentation when stronger fixation is considered necessary for OVFs with local kyphosis and angular instability. Pedicle screws (PSs) are widely used as an implant for posterior fixation, but given the risk of backing out in bones with severe osteoporosis, several measures have been taken to increase the strength such as by adding hooks. In cases of osteoporosis, hooks that can use cortical bone as an anchor are considered more useful than PS but are rarely used in minimally invasive surgery. We developed a minimally invasive posterior hook stabilization approach to directly stabilize the posterior spinal components as a new augmentation method for BKP and applied it to four cases of thoracolumbar OVF with neurological symptoms. The operation time was about 60 minutes, including BKP, and the estimated blood loss was about 10 ml. No postoperative implant problems occurred, and in all cases, neurological symptoms, such as buttocks and leg pain, were alleviated at an early stage after surgery. One patient had a postoperative adjacent vertebral body fracture that was conservatively treatable. Minimally invasive posterior hook stabilization, which we developed as a way of augmenting BKP, was considered useful for managing vertebral body fractures of the thoracolumbar spine with local kyphosis and angular instability.

## Introduction

Osteoporotic vertebral fractures (OVFs) have become a problem in elderly patients, reducing the quality of life, increasing systemic complications, and increasing case fatality [1]. Although many OVFs are treated conservatively, percutaneous cement augmentation, such as via balloon kyphoplasty (BKP) and vertebroplasty (VP), is often indicated for painful cases [2].

When OVF progresses, increased instability and nerve compression may cause a neurological deficit [3]. Various surgeries have been attempted to manage OVFs with inducing a neurological deficit, including anterior spinal fusion, posterior spinal fusion, posterior three-column osteotomy (e.g. pedicle subtraction osteotomy), and vertebroplasty with posterior spinal fusion [4-6]; however, the substantial surgical invasion involved remains an issue. Multiple OVFs can also cause severe sagittal imbalances and require surgical intervention [7].

Although the BKP procedure can support the anterior vertebral elements, the past literature previously reported that instability remains after BKP [8]. Spinal stability is ensured by the stability of each of the anterior and posterior elements. In flexion, the posterior components, such as the spinous process, interspinous ligament, and suprasupinatous ligament, play an important role [9-10]. When kyphosis progresses in OVFs, the elongation of the posterior ligaments may result in weakness against the flexion stability. Performing only BKP and VP for OVFs with severe kyphosis carries a risk of complication with adjacent fractures and paraparesis [11-12], although the risk of such complications can be mitigated by using pedicle screws (PSs) with either BKP or VP [13]. Lamina hook fixation has been reported to provide the same stability as PS and is often used in combination with a PS as posterior fixation [14-15]. Especially in cases of coarse bone, hooks with cortical bone as an anchor can be expected to be stronger than a PS, but few reports have described their use in minimally invasive surgery (MIS).

As a surgical treatment method for OVFs, we developed a minimally invasive posterior hook stabilization (MIPHS) approach to directly stabilize the posterior spinal components, and we applied it, as an augmentation of BKP, to four cases of OVF in the thoracolumbar spine with neurological deficit, with good results obtained.

## Technical report

BKP augmented with MIPHS was performed on OVFs of the thoracolumbar spine with neurological symptoms in four cases. Table 1 lists the age, sex, fracture site, cause of onset, preoperative waiting time, preoperative complications, preoperative neurological symptoms, canal stenosis of the lower hook attachment part, preoperative back pain visual analog scale (VAS), postoperative back pain VAS, inflated cement amount, operation time, estimated blood loss, postoperative complications, follow-up period, and osteoporosis treatment of all the cases.

**Table 1:** BKP augmented with minimal invasive posterior hook stabilization was performed on four OVF cases Preop, preoperative; Postop, postoperative; VAS, visual analog scale; BKP, balloon kyphoplasty; OVF: osteoporotic vertebral fracture

	Age	Gender	Fracture Site	Cause of Onset	Pre-op Waiting Time	Pre-op Complications	Preop Neurological Symptoms	Canal Stenosis (lower hook attachment part)	Preop Back Pain VAS	Postop Back Pain VAS	Inflated Cement Amount (ml)	Op Time (min)	Estimated Blood Loss (ml)	Postop Complications	Follow-up Period (month)	Osteoporosis Treatment
Case 1	81	Female	T12	Fall Down	12 weeks	Hypertension, Dementia, Stomach Cancer	Buttock Pain	none	90	10	10	65	10	None	5M	Romosozumab
Case 2	94	Female	L1	Fall Down	21 weeks	Hypertension, Dementia, Glaucoma	Drop Foot	none	80	20	10	60	10	None	3M	Teriparatide
Case 3	87	Female	T11	Fall Down	12 weeks	Hypertension, Ovarian cancer, Sjogren’s Syndrome	Buttock pain	none	80	20	12	67	5	None	6M	Teriparatide
Case 4	86	Male	T12	No Incentive	8 weeks	Decreased Cardiac Function, Kidney Cancer, Asthma	DropFoot, Leg Pain	none	100	10	14	50	10	Adjacent Vertebral Body Fracture	4M	Teriparatide

Osteoporosis treatment (e.g., teriparatide or romosozumab injection) was introduced in all cases.

The imaging assessment was performed using lateral X-ray films with the patient in the sitting and supine positions in order to assess the degree of angular instability of the fractured vertebrae (Table 2).

**Table 2:** Imaging findings, including local kyphosis, angular instability, and degree of nerve compression The focal kyphosis angle between the higher endplate of the upper vertebral body and the lower endplate of the lower vertebral body was measured. The difference between the angles in the sitting and supine positions was defined as angular instability. The ratio of occupation by bony fragments of the spinal canal was calculated as the ratio (as a percentage) of the bony fragment diameter to the adjacent normal canal diameter on mid-sagittal MR images, as previously reported.

	Preoperative Focal Kyphosis (degree)			Postoperative Focal Kyphosis (degree)			Occupation by Bony Fragments (%)
	Sitting	Supine	Angular Instability	Sitting	Supine	Angular Instability	
Case 1	57	41	16	42	33	9	NA
Case 2	41	17	24	34	13	21	40
Case 3	32	21	11	NA	NA	NA	28
Case 4	31	14	17	24	9	15	34

The focal kyphosis angle between the higher endplate of the upper vertebral body and the lower endplate of the lower vertebral body was measured as previously reported [8,16]. The ratio of occupation by bony fragments of the spinal canal was calculated as the ratio of the bony fragment diameter to the adjacent normal canal diameter on mid-sagittal MR images, as previously reported [5].

In this series, the preoperative kyphosis angle was 31-57º (average 40) and angular instability was 11-24º (average 17). The ratio of occupation by bony fragments of the spinal canal was 27.9-39.6 (average 33.5). The cement injection volume was 10-14 ml (12 ml on average). The operation time was 50-67 minutes (average 60 minutes), and the estimated blood loss was 5-10 ml (average 9 ml). The postoperative kyphosis angle was 24-42º (average 33º) and angular instability was 9-21º (average 15º). Preoperative back pain, buttock pain, and lower limb pain were alleviated shortly after surgery in all cases. No implant failure was observed during the follow-up period. While an adjacent fracture was found in one case after surgery, pain relief was obtained after conservative treatment.

**Figure 1:**
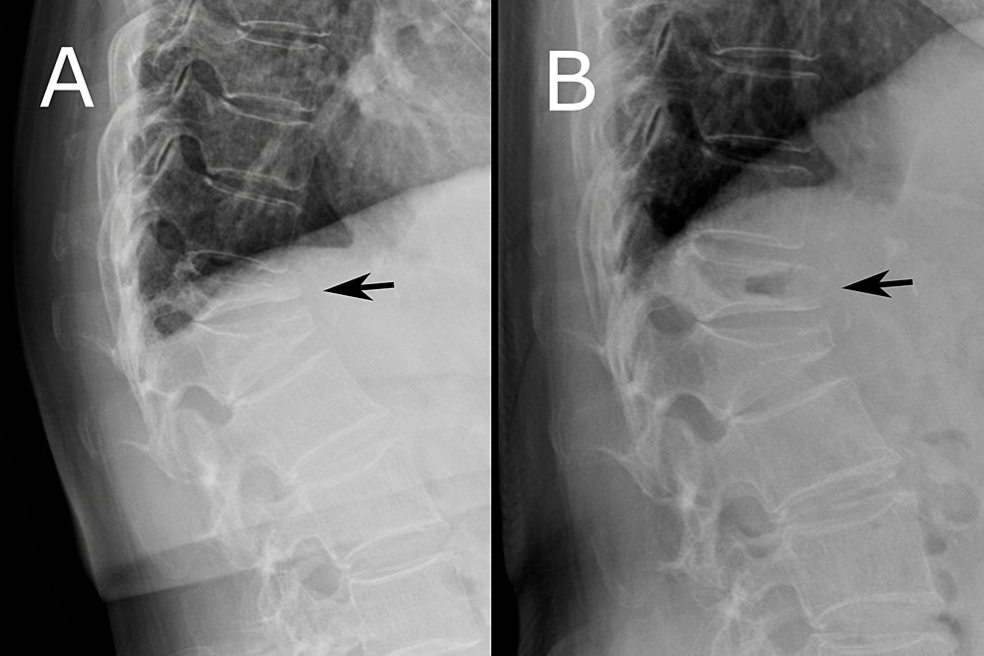
The surgical procedure of MIPHS as an augmentation of BKP is shown using a case of T12 OVF (Case 1 in Table 1) An 81-year-old woman who showed severe unstable OVF in T12 is shown. In the sitting position, the fractured vertebra completely collapsed and the height of the posterior wall was observed to have significantly decreased (A). However, in the supine position, the fractured vertebra is wide open and a cleft in the vertebra is observed (B). BKP, balloon kyphoplasty; OVF: osteoporotic vertebral fracture; MIPHS: minimally invasive posterior hook stabilization

**Figure 2:**
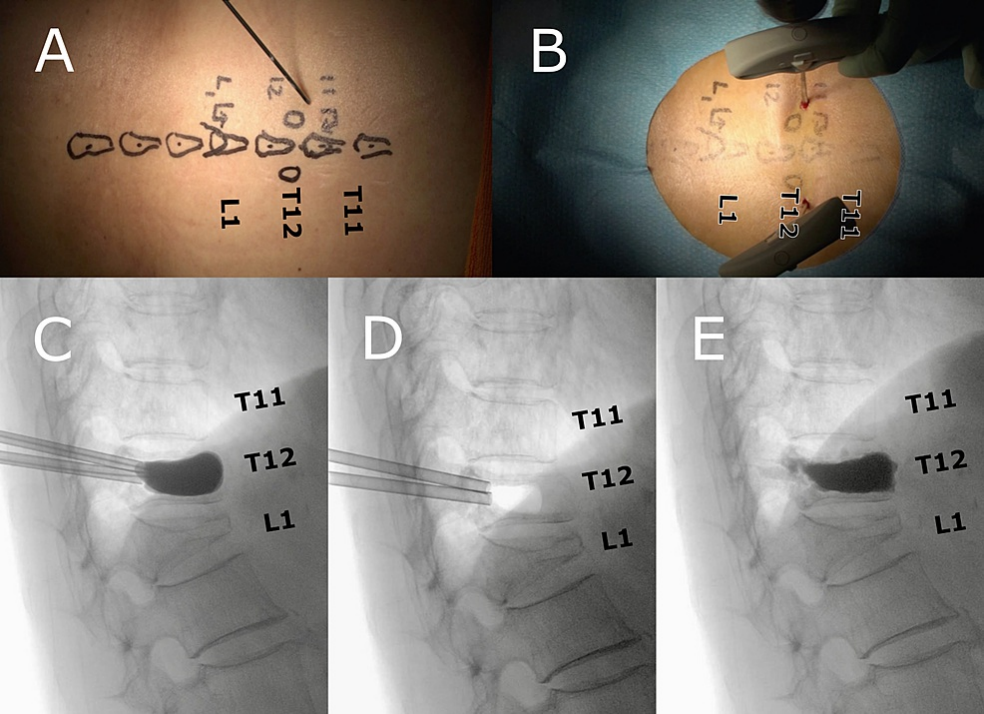
BKP reinforced the anterior vertebral element (Case 1 in Table 1) The location of the T12 pedicles, T11 spinous process, and L1 lamina were marked under fluoroscopy (A). Bone access needles were inserted into both the T12 pedicles. A balloon was inflated and the vertebral body height was confirmed (C). Bone cement was injected into the expanded space (D, E). BKP, balloon kyphoplasty

**Figure 3:**
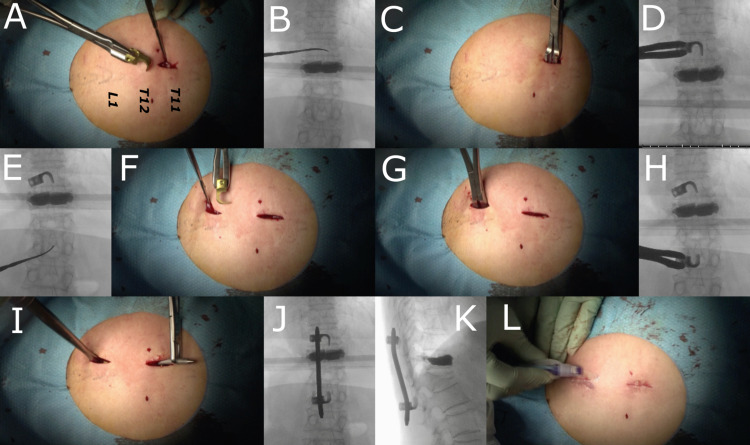
Minimal invasive posterior hook stabilization was performed (Case 1 in Table 1) The interspinous ligament at the upper end of the T11 spinous process was penetrated with a spatula (A, B). The hook was placed at the cranial surface of the T11 spinous process (C, D). A spatula was inserted beneath the L1 lamina (E, F). The hook caught the L1 lamina (G, H). After sliding the rod, attach the set-screws to the upper and lower hooks. Compress the holder between the upper and lower hooks with a compressor, but use only light force to avoid secondary fractures. Finally, fix the rod and hooks with set-screws (I, J, K, L).

The procedure of MIPHS as an augmentation of BKP is reported here using a case of T12 OVF (Video 1, Figures 1-3).

**Video 1:** Minimally invasive posterior hook stabilization in combination with balloon kyphoplasty (BKP) for osteoporotic vertebral fracture (OVF) Minimally invasive posterior hook stabilization, which we developed as a way of augmenting BKP, was considered useful for managing vertebral body fractures of the thoracolumbar spine with kyphosis.

The patient was placed in the prone position. BKP (Kyphon Balloon Kyphoplasty: Medtronic Sofamor Danek USA, Inc.) was performed under fluoroscopy. The balloon was inserted and expanded in the fractured vertebra, and a sufficient amount of cement was injected. After the completion of BKP, a 2-cm longitudinal incision was made 1.5 cm laterally from the midline at the level of the upper adjacent spinous process of the fractured vertebra. The interspinous ligament at the upper end of the spinous process was penetrated with a spatula to establish the hook attachment position (Figures 3A-3B). The open hook (CD HORIZON HOOK, wide blade, large size: Medtronic Sofamor Danek USA, Inc.) was placed face-down at the cranial surface in order to catch the spinous process under fluoroscopy (Figures 3C-3D). The hook is inserted from the side; however, it is possible to insert the hook without difficulty due to the kyphosis. If you use a small size hook, there will be insufficient play, which makes it difficult to connect the insertion procedure and the connecting rod. Because of the play with a large size hook, the hook can be inserted without any problem. Since the width of the lamina on the cranial side is narrow, it is difficult to attach the lamina hook with a small skin incision. Therefore, we decided to use a spinous process hook on the cranial side.

Then, a 2-cm longitudinal incision was made 1.5 cm laterally from the midline at the level of the lower edge of the inferior adjacent lamina of the fractured vertebra. The muscles were again separated to enable identification of the lower edge of the lamina, and a spatula was inserted beneath the lower end of the lamina as the hook attachment position (Figures 3E-3F). The large size hook was inserted in an upward position to catch the lamina under fluoroscopy (Figures 3G-3H). We attach the lower hook to the lamina instead of at the caudal surface of the spinous process. This is because when trying to attach it at the caudal surface of the spinous process, it is difficult to insert it from the side, and it is also difficult to fasten the rod and hooks by the setscrews from the side. The cranial side of the rod was bent to the ventral side to avoid skin protrusion at the proximal end of the rod, and the caudal side was slightly bent to the dorsal side to fit the lamina curve. After sliding the rod, attach the set screw to the upper and lower hooks (Figure 3I). Compress the holder between the upper and lower hooks with a compressor but only use light force to avoid secondary fractures. Finally, fix the rod and hooks with the set screws. The fascia and skin were then sutured to complete the surgery (Figures 3J, 3K, 3L).

**Figure 4:**
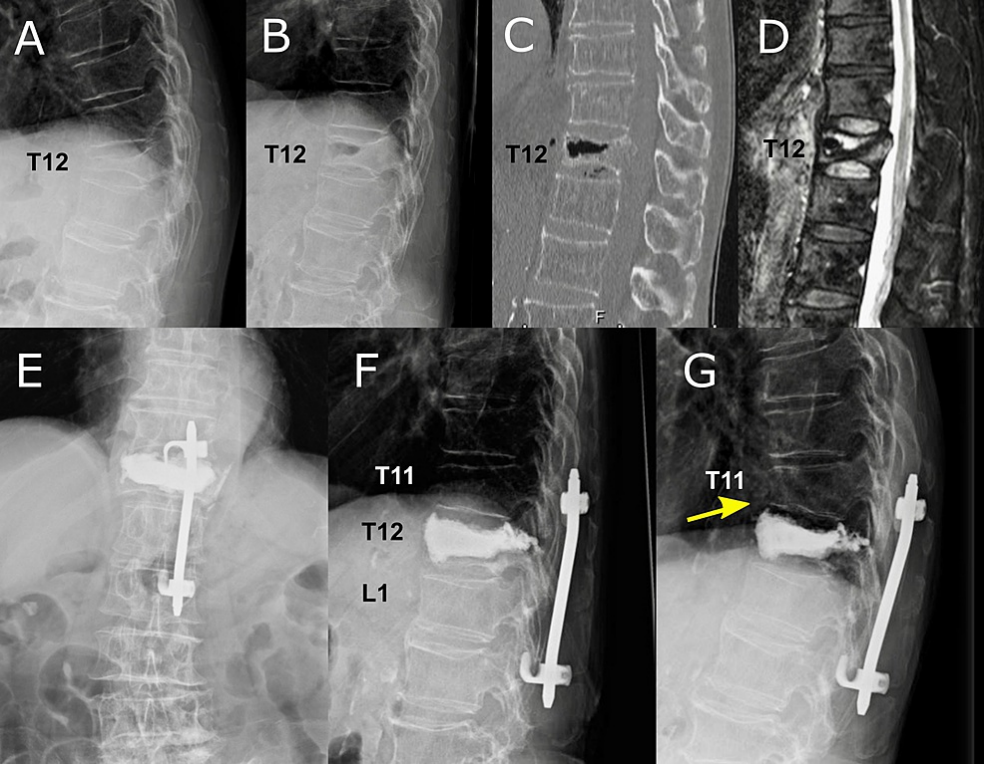
Another case of OVF treated by BKP with minimally invasive posterior hook stabilization (Case 4 in Table 1) An 86-year-old man showing severe instability of T12 OVF (A: sitting position, B: supine position). Computed tomography showed a vacuum cleft in the fractured vertebra (C). Magnetic resonance imaging showed a very high signal intensity in the T12 vertebra (D). BKP augmented with minimal invasive posterior hook stabilization was performed (E, F). He suffered an adjacent fracture of T11 (G: yellow arrow) but it healed successfully after conservative treatment. BKP, balloon kyphoplasty; OVF: osteoporotic vertebral fracture

We herein report another case of BKP augmented with MIPHS for the treatment of a fracture of the thoracolumbar spine (Figure 4, Case 4 in Table 1).

An 86-year-old man developed a T12 compression fracture without any special activities. He underwent conservative therapy for eight weeks with a brace. However, he gradually developed buttocks and posterior thigh pain and became unable to walk, at which point he was referred to our hospital. We initially planned pedicle subtraction osteotomy but found an impaired cardiac function (ejection fraction 30%), which made highly invasive surgery difficult. He underwent BKP augmented with MIPHS for symptom relief. The operation time was 50 minutes, and the estimated blood loss was 10 ml. His postoperative leg pain was alleviated, and he was able to walk and was discharged home. He suffered an adjacent fracture two weeks after surgery but healed with conservative treatment.

## Discussion

In thoracolumbar OVFs of the elderly, especially when accompanied by kyphosis, especially local kyphosis of >30º, the results of BKP alone may be poor [11]. Therefore, some measures have been taken such as using a percutaneous PS (PPS) together with BKP [13]. However, the PPS often backs out in the kyphosis, and skin damage due to implant protrusion becomes a problem. Furthermore, it is often necessary to increase the fixation range of PPS in order to increase the fixation strength.

In unstable OVFs, both the anterior and posterior elements of the spinal column are considered dysfunctional. Since BKP is considered able to provide strong anterior support, a certain amount of cement is required. Indeed, there are reports that it is better to increase the cement volume to ≥4.5 ml in order to obtain good results after BKP [17]. In our cases, the cement volume was quite high, at 12 ml on average, probably because the vertebral body was unstable and more strongly compressed than that observed in typical BKP cases.

The posterior component of the spine is thought to be important for flexion stability [9-10]. Our newly developed MIPHS method may inhibit extreme kyphosis by directly stabilizing the posterior spinal element. The MIPHS method can be performed using only two 2-cm skin incisions. The operation time was 60 minutes, including BKP, and the estimated blood loss was about 10 ml. The use of a skin incision avoiding the midline is advantageous for wound healing in cases with kyphosis. In addition, the relatively slight protrusion of the implant under the skin can reduce skin disorders.

Tarukado et al. previously reported that instability remains after BKP [8]. Ohba et al. reported that instability of the treated vertebrae after BKP caused paraparesis [10]. Hoshino et al. reported that the factors significantly contributing to neurological deficits in OVFs were angular instability of >15° and a ratio of occupation by bony fragments of the spinal canal of >42% [5]. The acquisition of stability of OVF can be expected to reduce postoperative adjacent fractures and nerve damage. Based on the above, OVF with an angular instability of ≥15°, a kyphosis angle of ≥30° degrees or more, and a spinal canal occupancy rate of ≥42% is considered to be at high risk, even if treated with BKP alone. MIPHS is not a way to completely fix the spine. Therefore, among these cases that are difficult to manage with BKP alone, it is considered that there are cases that are suitable for the application of MIPHS where nerve compression is not strong and paralysis is alleviated in the supine position.

The thoracolumbar spine seems to be a good indication of MIPHS. If it is used in a lower lumbar spine where lordosis exists, there is a risk that the hook will detach when the lordosis increases. Furthermore, it is not appropriate to apply MIPHS to a part with lordosis for the purpose of stabilizing kyphosis. Since the spinous process hangs down, it is considered difficult to install a hook on the spinous process in the middle thoracic spine. Given the above, MIPHS seems well-indicated when used to augment BKP in the thoracolumbar spine such as in cases of T11, T12, or L1 vertebral body fractures.

If BKP augmented with MIPHS is performed for OVF in a patient with neurological deficit and the postoperative neurological symptoms worsen, it may be necessary to add an additional two-stage surgery such as decompression or fusion surgery. Neuropathy with OVFs develops in both nerve compression and instability. BKP augmented with MIPHS is performed for the purpose of gaining stability with minimal invasiveness. If postoperative neurological symptoms do not improve as intended by gaining stability alone, the preoperative nerve compression may have been strong. If decompression surgery is added after BKP augmented with MIPHS, laminectomy of the fractured vertebral body is required, but resection of the upper lamina needs to be minimized in order to prevent an upper spinous process fracture, which is the anchor site of the superior hook.

Other potential risks associated with MIPHS include the risk of developing a new spinous process fracture after surgery and the possibility of implant dislocation after surgery. Since the use of a lamina hook may exacerbate central canal stenosis, it should be avoided in cases where central stenosis can be confirmed by MRI. The hook is placed in the interspinous ligament on the cranial side, which may also weaken the posterior support element in this area and cause local kyphosis.

It is considered that MIPHS is not indicated in the following situations: if the spinous process cannot be used as a hook anchor due to fracture, if it is difficult to attach the hook due to interspinous bone fusion, if it is clear that the entire spine alignment is very poor and short-range fixation cannot be maintained, or if paralysis occurs with a slight increase in instability due to high nerve compression at the fracture site.

The limitations of this study are the small number of patients and the short follow-up period. It is necessary to reconsider these points in the future.

In our series, the preoperative symptoms were all relieved after the operation. A postoperative adjacent fracture occurred in one case, but the original goal of improving pain and the neurological symptoms were obtained and the adjacent fracture was healed by conservative treatment. We believe that our MIPHS is useful as an augmentation procedure of BKP. In particular, its application is considered effective in cases of a thoracolumbar spine with kyphosis and angular instability. Regarding the usefulness for cases with neurological deficits, since the number of cases was quite small in this series, it will be necessary to consider this point again in the future.

## Conclusions

We developed a minimally invasive posterior hook stabilization approach as a new BKP reinforcement method for thoracolumbar osteoporotic vertebral fractures with kyphosis. Minimally invasive posterior hook stabilization was useful for managing cases of thoracolumbar osteoporotic vertebral fractures with kyphosis and angular instability. We believe that this method, which enables the use of hooks that were not previously used in minimally invasive surgery, may be a viable new option for posterior spinal augmentation. Since the number of cases was small and the follow-up period was relatively short in this series, it will be necessary to consider these points again in the future.
